# Combined effects of diet and genetic predisposition on kidney function outcomes

**DOI:** 10.1093/ckj/sfag196

**Published:** 2026-06-19

**Authors:** Giulia Barbieri, Ryosuke Fujii, Michele Filosi, Dariush Ghasemi-Semeskandeh, Martin Gögele, Giovanni Malerba, Maria Elisabetta Zanolin, Lucia Cazzoletti, Peter P Pramstaller, Cristian Pattaro, Essi Hantikainen

**Affiliations:** Institute for Biomedicine, Eurac Research, Bolzano/Bozen, Italy; Unit of Epidemiology and Medical Statistics, Department of Diagnostics and Public Health, University of Verona, Verona, Italy; Department of Preventive Medical Sciences, Fujita Health University School of Medical Sciences, Toyoake, Japan; Institute for Biomedicine, Eurac Research, Bolzano/Bozen, Italy; Institute for Biomedicine, Eurac Research, Bolzano/Bozen, Italy; Department of Human Genetics, Leiden University Medical Center, Leiden, The Netherlands; Integrative Data Analysis Unit, Health Data Science Centre, Human Technopole, Milan, Italy; Institute for Biomedicine, Eurac Research, Bolzano/Bozen, Italy; Department of Neurosciences, Biomedicine and Movement Sciences, University of Verona, Verona, Italy; Unit of Epidemiology and Medical Statistics, Department of Diagnostics and Public Health, University of Verona, Verona, Italy; Unit of Epidemiology and Medical Statistics, Department of Diagnostics and Public Health, University of Verona, Verona, Italy; Institute for Biomedicine, Eurac Research, Bolzano/Bozen, Italy; Institute for Biomedicine, Eurac Research, Bolzano/Bozen, Italy; Institute for Biomedicine, Eurac Research, Bolzano/Bozen, Italy

**Keywords:** CHRIS study, chronic kidney disease, gene-diet interaction, genetic predisposition, polygenic score

## Abstract

**Background:**

Chronic kidney disease (CKD) is a significant global health concern. While diet is crucial to CKD prevention and management, the interaction between diet and genetic predisposition to CKD is underexplored. We conducted an exploratory investigation of the interplay between a polygenic score (PGS) for kidney function and dietary exposures in relation to glomerular filtration rate estimated based on serum creatinine (eGFRcrea) on 5717 generally healthy adult participants of the Cooperative Health Research In South Tyrol study.

**Methods:**

Dietary intake was assessed using the GA^2^LEN food frequency questionnaire, from which we derived dietary quality indices including the Mediterranean, Alternative Healthy Eating Index and Dietary Approaches to Stop Hypertension diets, and nutrients relevant to kidney function (proteins, potassium and phosphorus). Genetic predisposition to lower kidney function was quantified using a PGS derived from 131 single nucleotide polymorphisms whose risk alleles were associated with lower eGFRcrea and higher blood urea nitrogen. We fitted multivariable generalized additive models incorporating diet-by-PGS interaction terms to model nonlinear associations with eGFRcrea.

**Results:**

The PGS was linearly associated with eGFRcrea (estimated degrees of freedom, df = 1.00, *P* < .0001), regardless of dietary exposures. All nutrients showed significant linear negative associations with eGFRcrea, a pattern not observed for dietary indices. Nominally significant nonlinear interactions were observed between the PGS and both protein (df = 4.93, *P* = .0298) and phosphorus intake (df = 5.37, *P* = .0283).

**Conclusions:**

The association of dietary protein and phosphorus intake with eGFRcrea may vary by genetic predisposition to low eGFRcrea levels, with stronger dietary effects in those with a high genetic predisposition.

## Graphical Abstract

**Graphical Abstract:**
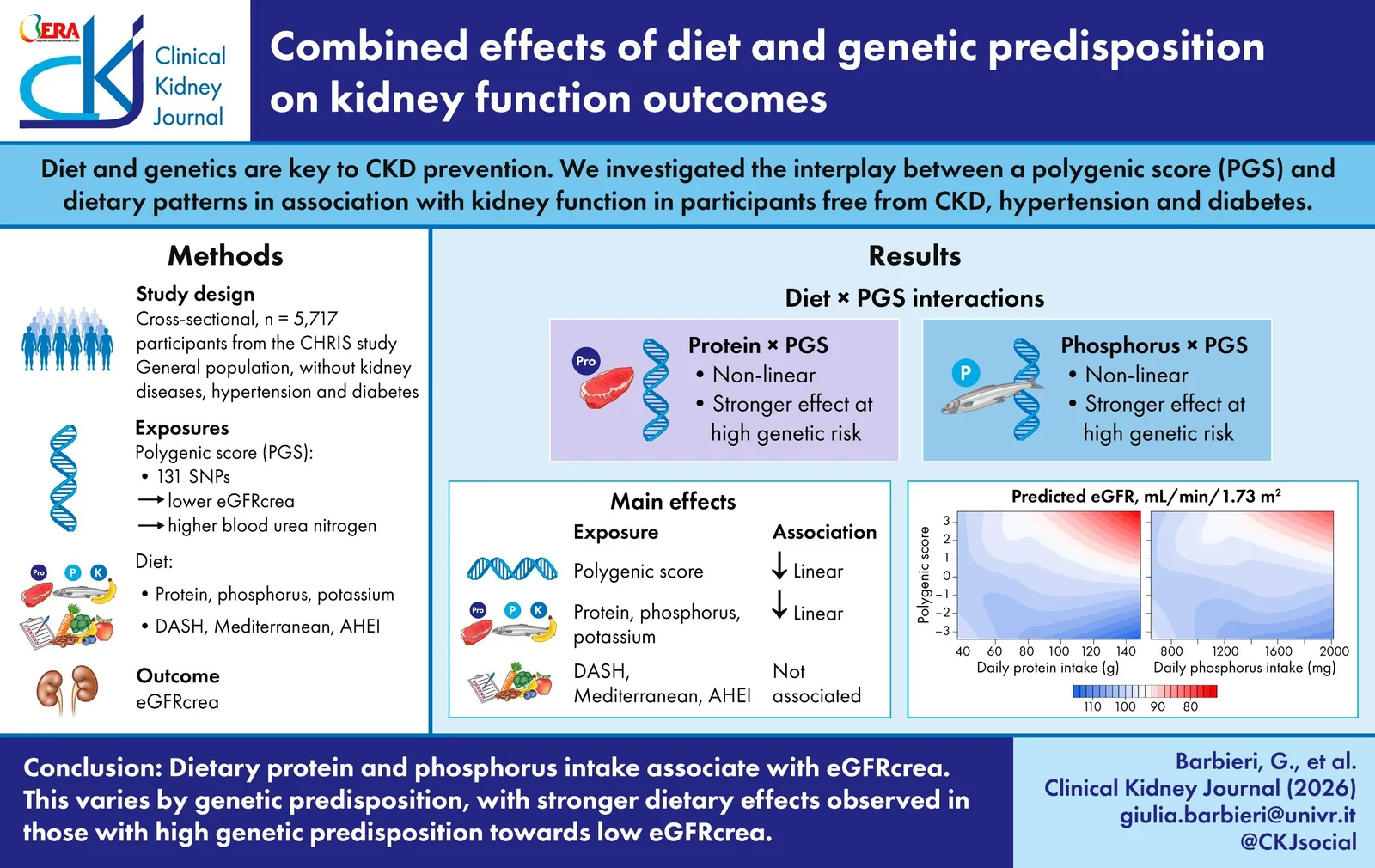

KEY LEARNING POINTS **What was known:**

- Chronic kidney disease (CKD) represents a global health burden and is predicted to become the fifth global cause of death by 2040. The development of CKD prevention strategies based on lifestyle modifications is urgently needed.
- While healthy dietary patterns have been associated with better kidney outcomes, the specific role of diet in CKD prevention remains inconclusive.
- Individual responses to diet are influenced by several factors of both genetic and lifestyle nature. However, the potential interplay between dietary factors and genetic susceptibility to CKD has been largely underexplored.

**This study adds:**

- This study shows that the relationship between dietary protein and phosphorus intake and kidney function may be modified by genetic risk, as measured by a polygenic score (PGS) for low kidney function.
- Nominally significant nonlinear interactions were identified, suggesting that individuals with a high genetic predisposition experience lower kidney function only when protein or phosphorus intake exceeds certain levels.

**Potential impact:**

- Individuals with high genetic risk for reduced kidney function may benefit from avoiding excessive intake of protein and phosphorus.

## INTRODUCTION

Chronic kidney disease (CKD), defined as the loss of kidney function over time, is a major health concern. CKD is a risk factor for end-stage kidney disease [1], cardiovascular disease morbidity [2], and all-cause mortality, and predicted to become the fifth global cause of death by 2040 [3]. CKD being strongly age-related, population ageing poses an urgent need to develop CKD prevention strategies based on lifestyle modifications as an inevitable path from both the medical and the socioeconomic perspective [4].

The concept of “food as medicine” has recently gained interest as healthier diets may help manage chronic diseases like CKD by addressing metabolic complications [5], inflammation, and gut health [6]. Nutritional modifications, such as protein intake restriction, play a key role in CKD management, however, dietary recommendations for CKD primary prevention are lacking [4]. Growing evidence shows that following healthy dietary patterns (DPs) might improve kidney function in generally healthy individuals. The Mediterranean diet and the Dietary Approaches to Stop Hypertension (DASH) diet, as well as higher consumption of plant-based foods and lower consumption of processed foods, have been linked to lower incident CKD risk [4, 7, 8]. Moreover, higher dietary potassium and lower dietary sodium intake are associated with decreased odds of CKD onset [4]. For protein intake, findings are still inconclusive with regard to CKD prevention [9]. Individual responses to diet are influenced by several factors of both genetic and lifestyle nature [10]. In the past decade, genomic studies demonstrated that genetic variations may interact with nutrients, foods, or DPs, affecting human health, including cardiovascular, metabolic, and oncologic diseases [10, 11]. The fact that effects of specific nutrients and DPs may vary based on individual genetic characteristics, such as metabolic function, might potentially explain some of the heterogeneous findings observed in studies investigating diet and CKD prevention. However, the interaction between genetic background and dietary exposures has been minimally explored in general population studies to date. Recently, potential interactive effects of a polygenic score (PGS) and DPs on the risk of kidney dysfunction were shown in a Korean population-based cohort [12]. Another study found no association between the Dutch dietary guidelines and kidney function in post-myocardial infarction patients, irrespective of genetic risk [13].

Using data from a generally healthy population sample, we previously demonstrated that higher protein intake from animal-based sources was associated with lower kidney function [14], while healthier DPs, such as the DASH diet, were associated with higher creatinine-based estimated glomerular filtration rate (eGFRcrea) [15]. Here we estimated whether genetic predisposition to reduced kidney function may moderate the relationship between dietary intake and eGFRcrea in generally healthy individuals. Within the Cooperative Health Research in South Tyrol (CHRIS) study [16], we analyzed several diet quality indices such as the DASH diet, Mediterranean diet, and Alternative Healthy Eating Index (AHEI). The DASH diet was included due to the substantial connection between hypertension and CKD, and the overlap among lifestyle recommendations [17]. The AHEI was chosen as a comprehensive measure of adherence to dietary recommendations known to reduce chronic disease risk [18]. The Mediterranean diet was included for its strong association with cardiovascular health and longevity, which are highly relevant in CKD, where cardiovascular disease is a leading cause of mortality [19]. In addition to these indices, we focused on specific nutrients such as protein, potassium, and phosphorus, which were chosen in advance based on their established physiological relevance in kidney function and their central role in clinical dietary management of CKD.

Dietary protein, potassium, and phosphorus cause heavy workload on the kidney. Higher protein intake increases nitrogenous waste, resulting in a heavier excretion effort, with potential effects in terms of raised blood flow and hyperfiltration. Phosphate handling is one of the kidney’s main roles. The kidney is also the primary regulator of potassium levels, to prevent hyperkalemia. Being modifiable, all three nutrients are potentially actionable. Other nutrients were not considered as their food frequency questionnaire (FFQ)-based quantification is either unreliable (e.g. sodium intake) or particularly prone to confounding (e.g. fat quality).

To prevent confounding due to creatinine metabolism, genetic predisposition to lower kidney function was assessed through a PGS based on genetic variants identified from a large genome-wide association study meta-analysis (GWAMA) that were associated with eGFRcrea and blood urea nitrogen (BUN) in a direction-consistent manner [20]. While being specific to kidney function, this PGS showed an equivalent association with CKD than PGS based on a larger number of markers based on eGFRcrea only [20]. Selecting variants jointly associated with eGFRcrea and BUN has been shown to be an efficient strategy to demonstrate the causal effect of lower kidney on higher blood pressure [21].

We explored linear and nonlinear effects of diet-PGS interactions on eGFRcrea, outlining the potential of genetic predisposition to impact associations between diet and kidney function.

## MATERIALS AND METHODS

### Study design

The CHRIS study was conducted between 2011 and 2018 and is detailed elsewhere [16, 22]. Briefly, study participants underwent blood drawing, urine collection, anthropometric assessments, blood pressure measurements, and clinical evaluations, in the early morning after an overnight fast. They also completed standardized self-administered and interviewer-administered questionnaires on their medical history and lifestyle.

Serum creatinine was measured using the Jaffé method with Roche Modular PPE and Abbott Diagnostic Architect c16000 instruments [23]. The eGFRcrea was estimated using the 2021 CKD-EPI equation based on serum creatinine [24], using the “*nephro*” R package v1.3 [25].

Participants with self-reported CKD, hypertension, or type 2 diabetes were excluded from the analysis. CKD was assessed using an ad-hoc kidney health questionnaire [26]. Self-reported hypertension and diabetes were defined either by reporting the condition directly or by indicating the use of medications corresponding to the relevant Anatomical Therapeutic Chemical (ATC) codes, which are listed in Supplementary Table S1. These exclusions were applied to minimize confounding by pre-existing kidney dysfunction and related comorbidities.

### Assessment of dietary exposures

Dietary intake was assessed using a self-administered version of the Global Allergy and Asthma European Network (GA^2^LEN) FFQ [27]. Participants were asked to report how often, on average, they consumed one portion of each of 229 foods and beverages over the last year, selecting among the following response options: rarely or never; once per month; once per week; 2–4 times per week; 5–6 times per week; once per day; and 2+ times per day. The consumption frequencies of each item were converted to grams per day, considering standard portion sizes. The most recent edition of the Composition of Foods Table [28] was used to further determine macro- and micro-nutrient and total energy intake (TEI; kcal/day).

All dietary exposures, including both individual nutrients and predefined dietary indices, were selected *a priori* based on established evidence linking them to kidney function and broader cardiometabolic health, therefore, each association was evaluated within this confirmatory framework. We included protein, phosphorus, and potassium intake among the dietary exposures due to their known associations with kidney function [9, 29, 30]. Although sodium is a key nutrient in the context of kidney health, we did not include sodium intake in our analyses because FFQ–based sodium estimates are known to be highly unreliable, largely due to systematic underestimation of sodium added during food processing and preparation. In addition, prior analyses in this cohort did not show an association between sodium intake and kidney function [31].

In addition, to provide a comprehensive investigation of healthy dietary recommendations, we decided to include three pre-defined dietary indices: the DASH-score [32], the AHEI-score [18], and Mediterranean-style (Med-score) [19]. The methods for calculating these indices are detailed in Table 1. All nutrient variables and the dietary components included in the calculation of the diet quality indices were adjusted for sex–specific energy intake using the residual approach before index computation [33].

**Table 1:** Summary of dietary indices, their components, and scoring methods.

Dietary indices	Healthy components	Unhealthy components	Scoring method	Score range*
DASH-score [29]	Fruits, vegetables, whole grains, low-fat dairy products, nuts, legumes	Red and processed meats, sodium, sugar-sweetened beverages	Sex-specific quintiles scored from 1 to 5 for “healthy” components and 5 to 1 for “unhealthy” components. SSB scored from 3 to 1 using sex-specific tertiles.	8–37
AHEI-score [17]	Fruits, vegetables, whole grains, nuts and legumes, omega-3 fatty acids, polyunsaturated fatty acids (PUFA), moderate consumption of alcohol	SSBs, red/processed meat, trans-fat, sodium, heavy consumption of alcohol	Score of 10 for perfect adherence and 0 for nonadherence. Proportional scoring between >0 and <10 for intermediate adherence.	0–110
Med-score [18]	Vegetables, fruit, legumes, cereals, and fish and the ratio of monounsaturated and polyunsaturated fatty acids to saturated fatty acids	Dairy, meat	Beneficial components scored as 1 if intake at or above sex-specific median, otherwise 0. Detrimental components scored in reverse.	0–9
	Exception: Alcohol intake		Alcohol from 10 to 50 g/d for men and from 5 to 25 g/d for women received a score of 1 and 0 otherwise	

*Score range: minimum∼no adherence, maximum∼perfect adherence.

### Polygenic score estimation

**Figure 1:**
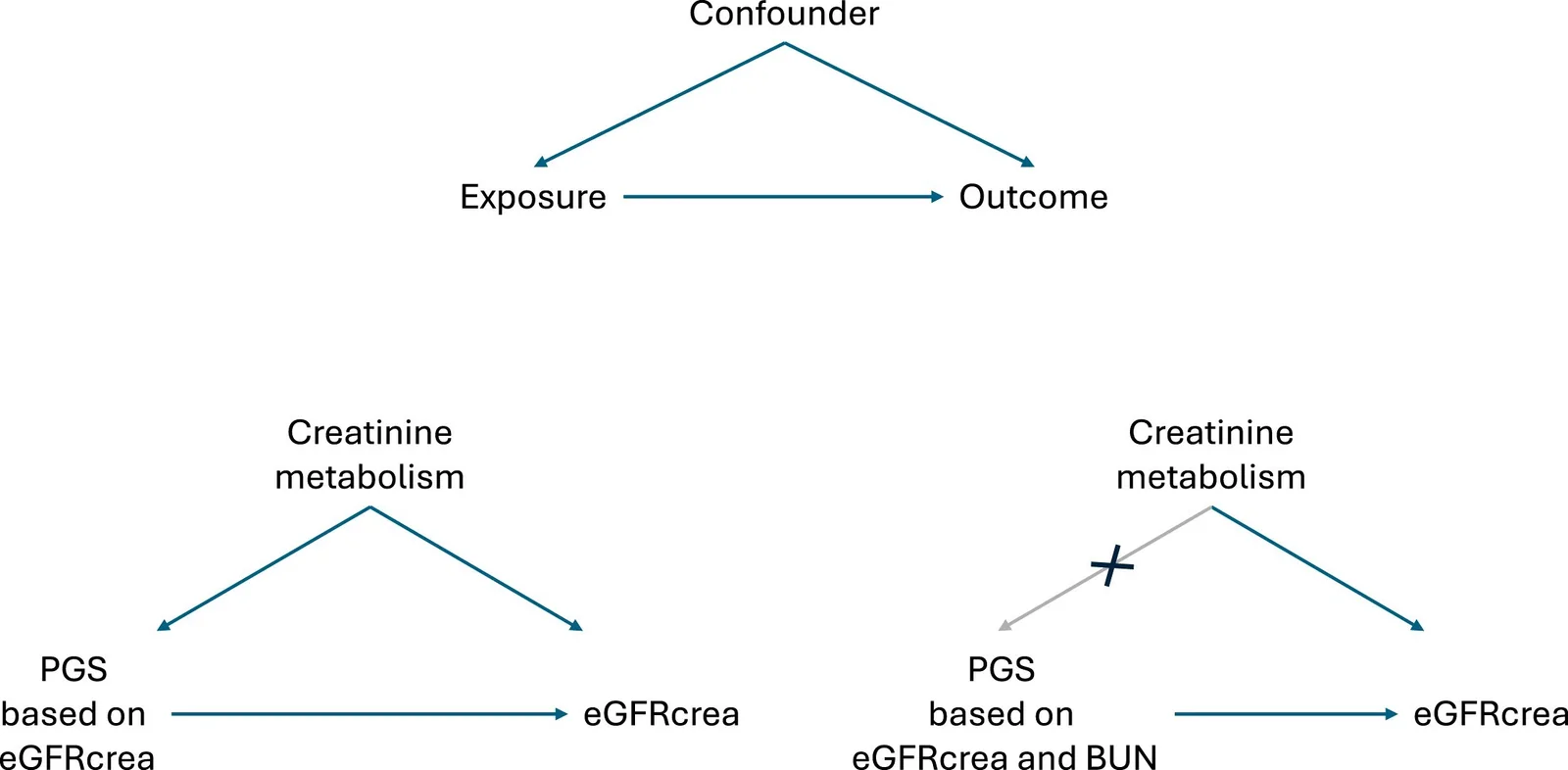
Conceptual diagram showing how, when modelling an association between a kidney function marker and a polygenic score (PGS), estimating the PGS using SNPs that are jointly associated with eGFR and BUN may prevent confounding by creatinine metabolism loci, even when kidney function is estimated based on serum creatinine only.

We estimated a PGS for each participant, using genotype data imputed on the Haplotype Reference Consortium (HRC) panel v1.1 (genome build GRCh37), including high-quality single nucleotide polymorphisms (SNPs) with an imputation score Rsq >0.9 and minor allele frequency, MAF, >0.5%. Effect sizes were derived from a large European-ancestry GWAMA of eGFRcrea on 765 348 individuals from the CKDGen consortium [20], after removal of the 4661 CHRIS samples included in the original GWAMA [34]. From the CKDGen GWAMA, we selected the 147 SNPs that showed evidence of kidney function relevance: that is, SNPs were associated with eGFRcrea at genome-wide statistical significance, plus the allele associated with lower eGFRcrea was also associated with higher blood urea nitrogen [20]. Among them, 131 SNPs were found in CHRIS: they all had high imputation quality (Table S2) and were all used for the PGS estimation, assuming an additive genetic model. Finally, the PGS was z-standardized and its sign inverted so that higher PGS reflects greater genetic risk for reduced kidney function (i.e. lower eGFRcrea). This targeted SNP selection approach was preferred over genome-wide or shrinkage-based PGS techniques to prioritize biological specificity for glomerular filtration and to limit the inclusion of loci related to creatinine metabolism, which could otherwise bias associations when using creatinine-based eGFRcrea as the outcome (conceptual framework shown in Fig. 1).

### Statistical analysis

Of all 13 393 CHRIS participants, we focused on those who were administered the FFQ (*n* = 8842). From these, we excluded: 34 participants with >20% missing FFQ items; 86 falling outside the 0.5^th^–99.5^th^ percentile range of the TEI/basal metabolic rate ratio; 5 with >80% missing biomarker data; 2081 reporting hypertension or diabetes based on questionnaires or medication use; (Table S1) 822 reporting a previous (*n* = 791) or uncertain (*n* = 31) kidney disease diagnosis [26]; 232 who were following a special diet; and 184 with low quality genotype data. This left 5717 participants available for statistical analyses (Fig. S1).

In this dataset, 99% of participants had less than 5% missing FFQ items, and no consistent pattern of missingness was identified across items. Highest missingness rates were observed for yogurt (2.53%), hard cheese (0.68%), veal (0.53%), and white rice (0.51%). All other items had <0.5% missingness. Given the very low proportion and random distribution of missing values in the FFQ data, we assumed that items missing represented zero intake, which is a commonly applied solution in nutritional epidemiology [35]. Other missing data were imputed using Multiple Imputation by Chained Equations (MICE) through the R package “*mice*” v3.14.0, generating five imputed datasets [36]. Sample characteristics were consistent between the imputed and unimputed datasets.

Correlations between dietary exposures (FFQ-derived nutrients, dietary scores) and the PGS were estimated as the Pearson’s correlation coefficient. The eGFRcrea variance explained by each exposure variable was quantified by the adjusted coefficient of determination (*R*²) following univariable linear model fitting.

The joint associations of diet and PGS with eGFRcrea were assessed via generalized additive models (GAMs) incorporating tensor product smooths, using the R package “*mgcv*” *v1.8-40*. First, we examined the effect of dietary exposures and PGS on eGFRcrea independently, using penalized regression splines (main effects models). Next, we developed models that incorporated both the main effects of diet and PGS, along with their interaction term (interaction models). GAMs allow estimating the degrees of freedom (df) of the nonlinear function used to model the relationship between predictor and outcome; df can be interpreted as the approximate degree of a polynomial that models the estimated association. A df close to 1 suggests a nearly linear relationship, whereas higher values indicate deviations from linearity. Since the main effects models indicated that most associations were linear (df = 1), we subsequently employed linear regression models (LMs) for those models considering main effects only. All models were adjusted for age, sex and the first 10 genetic principal components (PCs) based on common genotyped variants (MAF >5%) to account for family relatedness (minimally adjusted models). To control for potential residual confounding, we additionally included in the models physical activity (continuous Metabolic Equivalent of Task; MET) [37], smoking habits (Current, Former, Never smoker) [38], education (Primary school or no title; Lower secondary school; Vocational school; Upper secondary school; and University or higher), BMI (kg/m^2^), and TEI (kcal/day) (fully adjusted models). To prevent introducing collinear information in the fully adjusted model, we estimated the variance inflation factor using the *vif.gam* function of the R package “*mgcv*” *v1.8*-*40*. This analysis confirmed the absence of major collinearity problems (Table S3).

Potential overfitting was minimized using penalized regression splines with restricted basis dimensions, and model diagnostics indicated that overfitting was unlikely.

To facilitate interpretation of the PGS–exposure interactions, we present both heatmaps of predicted eGFR across the full range of exposures and PGS values, and predicted eGFR for the lowest (first tertile) and highest (third tertile) PGS groups, using the median PGS value within each tertile to generate marginal predictions from the GAM models.

Model diagnostics, including overfitting checks and Akaike Information Criterion values for all primary models (main effects and interaction models) are presented in Table S4.

As a sensitivity analysis, we fitted all models using an alternative PGS, estimated with PRS-CS [39] on genome-wide eGFR associations (PGS_eGFRcrea_). We now describe the correlation between the two PGSs and compare their associations with dietary exposures and eGFRcrea. Furthermore, to directly account for familial relatedness, as a sensitivity analysis we also fitted linear mixed–effects models for protein intake in which the genetic relatedness matrix defined the covariance structure of a kinship random effect. These results were compared with those from simple LMs adjusted and unadjusted for the first 10 genetic PCs. Finally, given the high correlation between phosphorus and protein, we tested the association with phosphorus after residualizing phosphorus for protein.

Given the hypothesis-testing framework was in large part confirmatory, statistical significance level was set at .05. However, investigations of interactions, concerning both their shape and significance, should be treated as explorative and *P*-values <.05 should still be interpreted with caution due to the absence of replication.

## RESULTS

Participants had a median age of 39.8 (IQR: 26.9, 51.6) years and 52.9% were females (Table 2). The median eGFRcrea level was 101.7 (IQR: 91.5, 112.0) ml/min/1.73 m². Participants had a median intake of 80.8 (IQR: 73.0, 89.1) g/day of protein, 3113.3 (IQR: 2781.3, 3502.6) mg/day of potassium, and 1261.3 (IQR: 1147.8, 1380.5) mg/day of phosphorus. The median DASH-score was 23.0 (IQR: 19.0, 27.0), the median AHEI-score was 63.6 (IQR: 54.3, 73.6), and the median Med-score was 4.0 (IQR: 3.0, 5.0).

**Table 2:** Description of the study sample (*N* = 5 717).

Variable group	Variable	Median (interquartile range)
Outcome	eGFRcrea, ml/min/1.73 m^2^	101.7 (91.5, 112.0)
Genetic exposure	PGS	0.0 (−0.7, 0.7)
Dietary exposure	DASH-score	23.0 (19.0, 27.0)
	AHEI-score	63.6 (54.3, 73.6)
	Med-score	4.0 (3.0, 5.0)
	Proteins, g/day	80.8 (73.0, 89.1)
	Potassium, mg/day	3113.3 (2781.3, 3502.6)
	Phosphorus, mg/day	1261.3 (1147.8, 1380.5)
Quantitative covariates	Age, years	39.8 (26.9, 51.6)
	Physical activity, MET-minutes/week	3516.0 (1584.0, 6312.0)
	Total energy intake, kcal/day	1894.1 (1555.0, 2310.0)
	BMI, kg/m^2^	24.3 (22.0, 27.2)
Variable group	Variable	N (%)
Categorical covariates	Sex	
	* Male*	2693 (47.1)
	* Female*	3024 (52.9)
	Smoking habit	
	* Never smoker*	3203 (56.0)
	* Current smoker*	1137 (19.9)
	* Past smoker*	1377 (24.1)
	Education	
	* Primary school or no title*	286 (5.0)
	* Lower secondary school*	857 (15.0)
	* Upper secondary school*	1564 (27.4)
	* Vocational school*	2376 (41.6)
	* University or higher*	634 (11.1)

The PGS was normally distributed (Fig. S2a) and uncorrelated with the dietary variables (Fig. S2b). Protein intake showed a strong positive correlation with phosphorus (*r* = 0.72), and low correlation with potassium and all dietary indices (Fig. S2b). Potassium showed maximal correlation with the DASH-score (*r* = 0.51). The three dietary indices were strongly correlated with each other (*r* > 0.58).

The PGS explained 1.46% of the eGFRcrea variance, while dietary exposures explained between 0.22% (phosphorus) and 2.23% (DASH-score) (Table S5).

In the main effect models with minimal adjustment, we observed a negative linear association between the PGS and eGFRcrea (GAM: df = 1.00, *P*-value < .0001, Table 3, LM: Coefficient = −1.72, *P*-value < .0001, Table S6). Linear associations with eGFRcrea were observed also for protein intake (GAM: df = 1.00, *P*-value < .0001; LM: Coefficient = −0.0490, *P*-value < .0001), potassium (GAM: df = 1.00, *P*-value = .022; LM: Coefficient = −0.0007, *P*-value = .0020) and phosphorus (GAM: df = 1.00, *P*-value = .0453, Table 3; LM: Coefficient = −0.0016, *P*-value = .0339, Table S6). No significant associations were observed with the dietary indices. Results remained unchanged in the fully adjusted model, except for phosphorus, for which the association with eGFRcrea became nonsignificant (Table S6).

**Table 3:** Results of the GAM analysis on the association of PGS and dietary exposures^a^ with eGFRcrea. Reported are the estimated degrees of freedom (df) and *P*-values for all estimated models.

	Minimally adjusted model*		Fully adjusted model**	
Main effects models^b^ ^b^	df	*P*-value	df	*P*-value
PGS	1.00	<.0001	1.00	<.0001
Proteins	1.00	<.0001	1.00	<.0001
Potassium	1.00	.0022	1.00	.0120
Phosphorus	1.00	.0453	1.00	.1502
DASH	2.11	.1183	1.94	.0609
AHEI	1.63	.2114	1.00	.5672
Mediterranean	1.00	.9474	1.00	.4064
Interaction models ^c^	df	*P*-value	df	*P*-value
PGS	1.00	<.0001	1.00	<.0001
Proteins	1.00	<.0001	1.00	<.0001
Proteins:PGS	4.93	.0298	4.82	.0335
PGS	1.00	<.0001	1.00	<.0001
Potassium	1.00	.0010	1.00	.0061
Potassium:PGS	1.00	.6783	1.00	.7064
PGS	1.01	<.0001	1.01	<.0001
Phosphorus	1.00	.0241	1.00	.0864
Phosphorus:PGS	5.37	.0283	5.46	.0223
PGS	1.00	<.0001	1.00	<.0001
DASH-score	2.22	.0606	2.13	.0246
DASH-score:PGS	1.01	.4705	1.01	.5379
PGS	1.00	<.0001	1.01	<.0001
AHEI-score	1.91	.1788	1.54	.3974
AHEI-score:PGS	1.04	.2773	1.01	.2688
PGS	1.00	<.0001	1.00	<.0001
Med-score	1.02	.8872	1.02	.2853
Med-score:PGS	1.00	.2548	1.01	.2187

*Abbreviations*: GAM, generalized additive model; PGS, polygenic score; eGFRcrea, estimated glomerular filtration rate; df, degrees of freedom.

*Adjusted for: age, sex, and the first 10 genetic principal components (PCs) of the genotyped data.

**Adjusted for: age, sex, first 10 genetic PCs of the genotyped data, body mass index, physical activity, smoking habits, and education.

a *Dietary exposure*: Proteins, Potassium, Phosphorus, AHEI, Mediterranean, DASH.

b Each term was included in a separate, minimally adjusted and fully adjusted models.

c Main effects and their interaction were included in the minimally adjusted and fully adjusted models.

In the interaction models with minimal adjustment, none of the dietary indices exhibited a significant interaction with the PGS (DASH-score/PGS: df = 1.01, *P* = .4705, AHEI-score/PGS: df = 1.04, *P* = .2773; Med-score/PGS: df = 1.00, *P* = .2548, Table 3). Among the nutrients, protein and phosphorus intake showed nominally significant nonlinear interactions with the PGS (protein-PGS: df = 4.93, *P* = .0298; phosphorus-PGS: df = 5.37, *P* = .0283), while no interaction was observed with potassium (potassium-PGS: df = 1.00, *P* = .6783). Results remained essentially the same when comparing the minimally adjusted (Table 3, Fig. S3a, b) to the fully adjusted models (Table 3, Fig. S4a, b). Results were also consistent with those obtained from kinship-based mixed regression models for proteins (Table S7).

**Figure 2:**
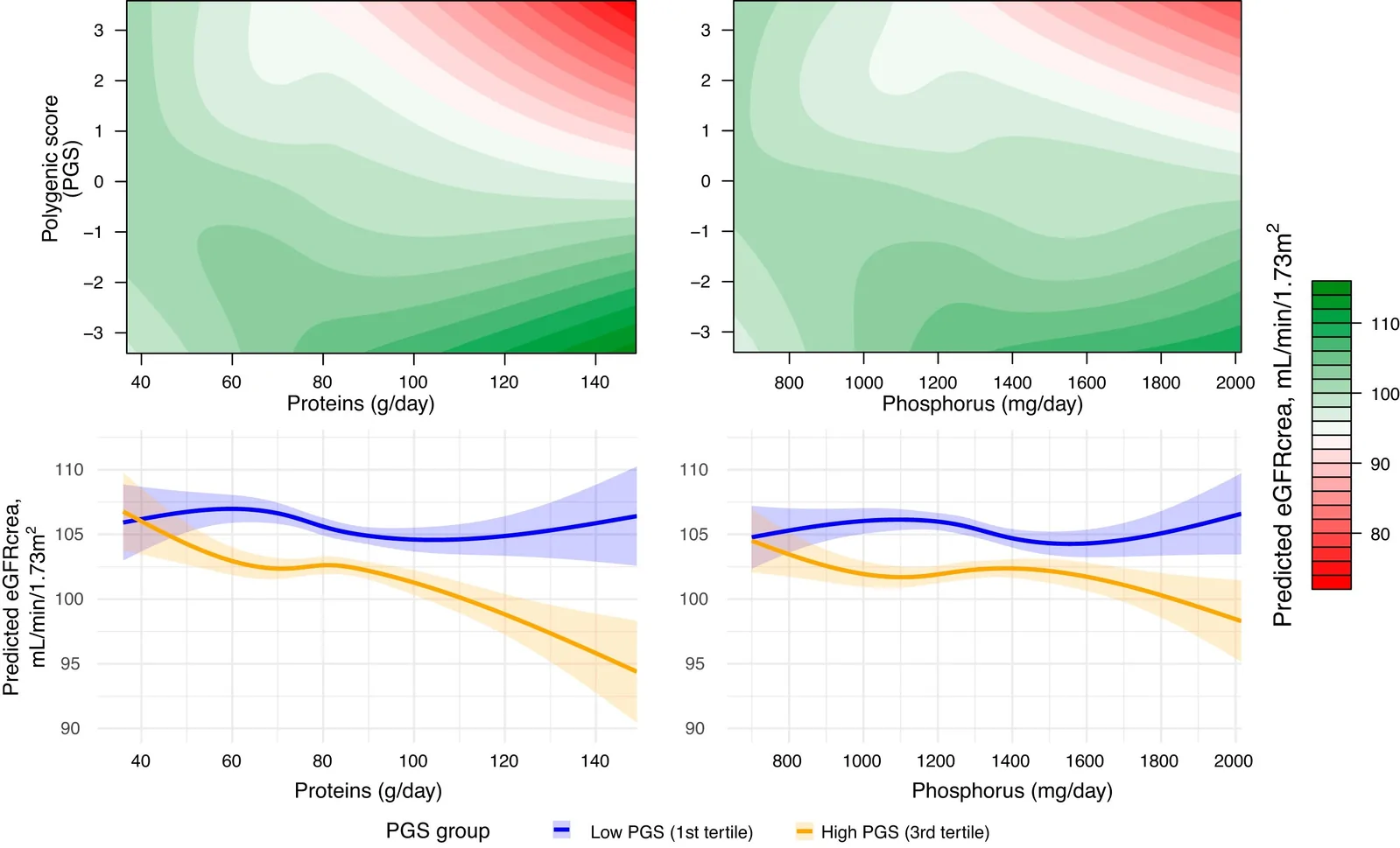
Predicted eGFRcrea levels by dietary exposure* and polygenic score (PGS), derived from minimally adjusted** GAM with nonlinear tensor product smooths. Upper panels: heatmaps of predicted eGFRcrea levels as a function of protein intake (left) and phosphorus intake (right) across PGS levels; the color gradient ranges from red (lower eGFRcrea) to green (higher eGFRcrea). Lower panels: predicted marginal effects of dietary exposures on eGFRcrea for individuals in the lowest (first tertile) and highest (third tertile) PGS groups; solid lines represent predicted eGFRcrea and shaded ribbons indicate 95% confidence intervals. Abbreviations: GAM, generalized additive model; PGS, polygenic score; eGFRcrea, estimated glomerular filtration rate. **Minimally adjusted: Adjusted for age, sex, and the first 10 genetic principal components (PCs) of the genotyped data.

The visual interpretation of the interaction results for protein and phosphorus in the minimally adjusted model (Fig. 2) suggested that both nutrients were associated with lower eGFRcrea in individuals with high PGS but not in those with low PGS. Despite the more mitigated effects of phosphorus, we observed very similar, almost overlapping, associations between the two nutrients and PGS, potentially reflecting the high correlation between them (Fig. S2b). Additionally, the nonlinear nature of these interactions indicated that a high PGS was associated with lower eGFRcrea levels only when protein and phosphorus intakes exceeded the median daily intake (protein > 80 g/day, phosphorus > 1260 mg/day). A complete graphical overview of all nonlinear associations is reported in Fig. S3a, b and Fig. S4a, b. Residualization analysis of phosphorus on protein, which we conducted to assess whether collinearity played any role given their strong correlation, showed that both the main effect and the phosphorus–PGS interaction lost statistical significance (Table S8).

When looking at the independent main effects of the PGS and diet in the interaction models, the PGS showed a linear association with eGFRcrea (df ≈ 1.00, *P* ≤ .0001), regardless of the dietary exposure (Table 3). Coherently to the main effects models, all the FFQ-derived nutrients, except phosphorus, were linearly associated with eGFRcrea (protein: df = 1.00, *P* < .0001; potassium: df = 1.00, *P* = .0010; phosphorus: df = 1.00, *P* = .0864), The DASH score was associated with eGFRcrea under a quadratic model (df = 2.22, *P* = .0606), with the association becoming significant when controlling for additional potential confounders (fully adjusted model: df = 2.13, *P* = .0246). All other results remained consistent in the fully adjusted model.

Sensitivity analyses showed that the PRS-CS–derived PGS based on creatinine-based eGFR only (PGS_eGFRcrea_) was positively correlated with our primary PGS based on eGFRcrea and BUN loci (Fig. S5) and explained a substantially larger proportion of variance (Table S5). Nevertheless, no meaningful differences were observed in the inferential results from model fitting. When using PGS_eGFRcrea_ in place of our primary PGS, we observed that PGS’s main effects changed from linear to nonlinear, while the interaction effects between PGS and dietary indices changed from nonlinear to linear, across all models (Table S9). However, in terms of statistical significance, results were unchanged, with one exception: the PGS_eGFRcrea_ -by-phosphorus interaction became not significant. This is likely due to a combination of the nonlinearity of the PGS_eGFRcrea_ effect on eGFRcrea combined with the minimal eGFRcrea variance explained by phosphorus (Table S5).

## DISCUSSION

We investigated the joint association of healthy dietary patterns, kidney health-related nutrients, and a kidney function PGS with eGFRcrea in a cross-sectional general population sample with no diagnosis of CKD, diabetes, and hypertension. We observed that higher protein and phosphorus intake were associated with lower kidney function in individuals with higher genetic predisposition toward lower eGFRcrea. We further observed a nonlinear association between the DASH diet and eGFRcrea, regardless of the PGS. No associations were observed between the potassium intake, AHEI and the Mediterranean diet and eGFRcrea, irrespective of the PGS. Our analysis may add new evidence about the interplay between genetic risk and diet in relation to kidney function, highlighting that high protein and phosphorus intake can be particularly unfavourable in people at high genetic risk.

Protein intake has gained significant attention in relation to CKD prevention, however current evidence has not reached a clear consensus on the effect of high-protein diets on kidney health in the generally healthy population [9]. Protein-rich diets can cause intraglomerular hypertension, possibly resulting in kidney hyperfiltration, glomerular injury, and proteinuria [9]. Intake of animal-sourced protein may harm the kidneys [14]. Phosphorus is central to the pathogenesis of CKD-mineral and bone disorder [40] and a low-phosphorus diet potentially alleviates CKD by reducing the levels of serum fibroblast growth factor 23 associated with CKD development and progression [41]. Here we observed that genetic predisposition may modify the association between dietary protein and phosphorus intake and kidney function in healthy individuals. To the best of our knowledge, only one other study has explored the gene-diet interaction on the risk of kidney dysfunction in a population-based cohort considering population-specific dietary patterns [12], while another recent study investigated associations between genetic CKD risk, Dutch dietary guidelines for CVD and kidney function decline among post-myocardial infarction patients. Our findings suggest that genetic predisposition for lower eGFRcrea may amplify the negative effect of high protein and phosphorus intake on kidney function. The observed nonlinearity implied that individuals with higher genetic risk were more susceptible to lower kidney function specifically when dietary intake of protein, and phosphorus, exceeded certain levels. Interestingly, for those individuals with lowest PGS and highest protein and phosphorus intake, we observed highest levels of eGFRcrea, suggesting that healthy individuals with a genetic predisposition to healthy kidney function might be able to keep up with a strong filtering capacity even at high levels of habitual protein intake. Nevertheless, given the cross-sectional design, we cannot exclude potential reverse causation. Also, previous population-based studies have shown that higher protein intake is associated with increased eGFR in cross-sectional analyses [42, 43], whereas longitudinal data suggest potential adverse effects [43]. Therefore, further research using longitudinal datasets should aim to assess how individuals with lower genetic predisposition are affected by higher protein intake. Because protein and phosphorus intake were analyzed in separate models, we conducted a residualization analysis of phosphorus on protein to assess whether collinearity played any role given their strong correlation. This analysis showed that after adjusting phosphorus for protein, both the main effect and the interaction of phosphorous with the PGS were attenuated and no longer statistically significant. This pattern suggests that the phosphorus findings may largely reflect shared variance with protein intake.

The small effect sizes involved (PGS explaining 1.4% and phosphorus 0.22% of eGFRcrea variance) limit the ability to detect additional independent effects, underscoring the need for further methodological refinement. Longitudinal studies, with well-established measures of renal outcomes are warranted to further evaluate how genetic risk may influence the association between diet and kidney function. In future, this approach could help inform personalized dietary recommendations.

In our investigation, we did not observe any interactions between dietary indices and PGS, however, we observed a u-shaped association between the DASH-score end eGFRcrea, irrespective of genetic risk. Interestingly, this association was only significant in models adjusting for the PGS, whereas borderline significant without PGS adjustment. Given the DASH has been developed to reduce hypertension, removing hypertensive participants from our analysis might have attenuated any association in our analysis. We previously reported a beneficial association between DASH diet and kidney function in the CHRIS cohort, however, this association was only observed in males [15]. Our results are in line with several prospective studies linking this diet to lower risk of developing kidney disease [17, 47], whereas one study found no associations with changes in eGFR, urinary albumin-to-creatinine ratio or incident CKD [48]. Although all DPs were strongly correlated, reflecting their alignment as healthy DPs, we did not observe any associations between the Mediterranean or the AHEI diet and eGFRcrea, which aligns with results reported by Missikpode *et al*. 2021 [48], but contrasts several other studies [49].

While our analysis presents various strengths, like the large sample size and the availability of a validated FFQ, several limitations must be acknowledged. First, we should recognize the exploratory nature of the interaction analyses, for which, nominally significant results should be interpreted with caution until independent replication is provided. Next, self-reported dietary intake is prone to recall bias and measurement error. By adjusting dietary variables for TEI, we not only control for confounding, but we also reduce some of the extraneous variation resulting from factors like body weight, physical activity and metabolic efficiency [50]. Imputation of all missing items as zero intake could have led to nutrient intake underestimation. However, an analysis of missing data distribution confirmed a low proportion of missing data as well as the absence of specific patterns of data missingness. Given the FFQ was delivered to participants’ homes prior to the assessments of any health conditions conducted at the study center, any remaining misclassification of the exposure variables is likely nondifferential, which would bias estimates of association toward the null [51]. The cross-sectional design prevents us from determining whether dietary habits precede differences in kidney function, and therefore no causal conclusions can be drawn. To reduce the risk of reverse causation, we restricted the analysis to individuals who self–reported no history of kidney disease, hypertension, or diabetes and who did not report following a specific diet for medical reasons. On the other hand, the decision to focus on healthy individuals may have introduced greater measurement error in the outcome, as the discrepancy between estimated and measured GFR tends to be more pronounced in healthy individuals compared to those with impaired kidney function [52]. When weighing the competing risks of collider bias, potentially leading to reverse causation if individuals with diabetes, hypertension, or CKD are included, against reduced clinical relevance due to increased measurement error, it is important to note that creatinine-based GFR estimates remain applicable to healthy individuals in the general population. For example, genomic studies of eGFR in the general population, which included a higher proportion of individuals with eGFR >90 ml/min/1.73 m², showed a notable enrichment of kidney-specific genes compared to genes expressed in other tissues [53]. Additionally, even modest reductions in eGFR can predict a greater risk of adverse outcomes in healthy individuals with elevated eGFR levels [54]. These findings support the overall validity of our analyses. Furthermore, at higher GFR levels, estimation errors do not appear to systematically overestimate or underestimate GFR [25]. Consequently, our results are likely biased toward the null, limiting the detection of additional significant findings that might emerge with larger sample sizes, while reducing the likelihood of false positive results. Another limitation is that kidney function could not be measured in CHRIS participants but only estimated based on serum creatinine, which may also reflect muscle mass metabolism and, consequently, might be influenced by short-term protein intake. However, utilizing 12-hour fasting plasma samples, as conducted in the CHRIS study, has been demonstrated to reduce the acute impact of meat consumption on serum creatinine levels [55]. We further mitigated the muscle-mass issue by selecting a PGS which only included genetic variants that were demonstrated to be relevant to kidney function: all variants used for the PGS estimation were associated with at least two different markers of kidney function, namely creatinine-based eGFR (eGFRcrea) and blood urea nitrogen from the CKDGen consortium [20]. As shown in other contexts [21], also in the present case, selecting fewer but more specific kidney function variants for PGS estimation did not cause any loss of results compared to a more sophisticated score based on all but less specific variants associated with eGFRcrea. It is worth noting that the exclusion of individuals with CKD, hypertension, and diabetes has probably prevented to observe the upper tails of the PGS distribution, which might be enriched of variants related to monogenic or nearly monogenic CKD forms, and where genetic predisposition may affect eGFR levels independently of dietary habits. Similarly, this prevented any observation in the lower tail of the eGFRcrea distribution. Finally, given that we used a European-ancestry-based PGS with application to a European ancestry population sample, generalizability of our findings to non-European populations may be limited.

## CONCLUSION

The association between dietary protein and phosphorus intake and eGFRcrea might be influenced by individuals’ genetic predisposition to low eGFRcrea levels, with stronger associations observed in individuals with high genetic predisposition. Although the observed associations were small, these findings suggest genetic variability as a potential source of heterogeneity underlying diet and health relationship, which should be considered in future studies.

## Supplementary Material

sfag196_Supplemental_File

## Data Availability

For safeguarding compliance with the CHRIS study participants’ consent and the applicable personal data processing legislation, data qualifying as personal data under the GDPR that have been used for this analysis, may only be shared according to the CHRIS Access Policy (https://chrisportal.eurac.edu/downloads).

## References

[1] Fox CS, Matsushita K, Woodward M et al. Associations of kidney disease measures with mortality and end-stage renal disease in individuals with and without diabetes: a meta-analysis. Lancet. 2012;380:1662–73. 10.1016/S0140-6736(12)61350-6 23013602 PMC3771350

[2] Fujii R, Melotti R, Köttgen A et al. Integrating multiple kidney function markers to predict all-cause and cardiovascular disease mortality: prospective analysis of 366 758 UK Biobank participants. Clin Kidney J. 2024;17:sfae207. 10.1093/ckj/sfae207 39135936 PMC11317837

[3] Foreman KJ, Marquez N, Dolgert A et al. Forecasting life expectancy, years of life lost, and all-cause and cause-specific mortality for 250 causes of death: reference and alternative scenarios for 2016-40 for 195 countries and territories. Lancet. 2018;392:2052–90. 10.1016/S0140-6736(18)31694-5 30340847 PMC6227505

[4] Kelly JT, Su G, Zhang L et al. Modifiable lifestyle factors for primary prevention of CKD: a systematic review and meta-analysis. J Am Soc Nephrol. 2021;32:239–53. 10.1681/ASN.2020030384 32868398 PMC7894668

[5] Tessier A-J, Wang F, Korat AA et al. Optimal dietary patterns for healthy aging. Nat Med. 2025;31:1644–52. 10.1038/s41591-025-03570-5 40128348 PMC12092270

[6] Mafra D, Borges NA, Lindholm B et al. Food as medicine: targeting the uraemic phenotype in chronic kidney disease. Nat Rev Nephrol. 2021;17:153–71. 10.1038/s41581-020-00345-8 32963366

[7] van Westing AC, Küpers LK, Geleijnse JM. Diet and kidney function: a literature review. Curr Hypertens Rep. 2020;22:14. 10.1007/s11906-020-1020-1 32016564 PMC6997266

[8] Mozaffari H, Ajabshir S, Alizadeh S. Dietary approaches to stop hypertension and risk of chronic kidney disease: a systematic review and meta-analysis of observational studies. Clin Nutr. 2020;39:2035–44. 10.1016/j.clnu.2019.10.004 31669002

[9] Ko GJ, Rhee CM, Kalantar-Zadeh K et al. The effects of high-protein diets on kidney health and longevity. J Am Soc Nephrol. 2020;31:1667–79. 10.1681/ASN.2020010028 32669325 PMC7460905

[10] Qi L . Nutrition for precision health: the time is now. obes. 2022;30:1335–44. 10.1002/oby.23448 35785484

[11] Stanzick KJ, Li Y, Schlosser P et al. Discovery and prioritization of variants and genes for kidney function in >1.2 million individuals. Nat Commun. 2021;12:4350. 34272381 10.1038/s41467-021-24491-0 PMC8285412

[12] Jang MJ, Tan LJ, Park MY et al. Identification of interactions between genetic risk scores and dietary patterns for personalized prevention of kidney dysfunction in a population-based cohort. Nutr. Diabetes. 2024;14:1–9. 10.1038/s41387-024-00316-z 39143076 PMC11325018

[13] van Westing AC, Heerkens L, Cruijsen E et al. Diet quality in relation to kidney function and its potential interaction with genetic risk of kidney disease among Dutch post-myocardial infarction patients. Eur J Nutr. 2024;63:1373–85. 10.1007/s00394-024-03355-5 38430449 PMC11139691

[14] Vukovic V, Hantikainen E, Raftopoulou A et al. Association of dietary proteins with serum creatinine and estimated glomerular filtration rate in a general population sample: the CHRIS study. J Nephrol. 2022;36:103–14. 10.1007/s40620-022-01409-7 35930180 PMC9894942

[15] Barbieri G, Garcia-Larsen V, Lundin R et al. DASH diet, reduced rank regression dietary patterns and relations with kidney function in the CHRIS general population study. BMC Nephro. 2026 Apr 20;27:347. 10.1186/s12882-026-04896-z PMC13224596 42010532

[16] Lundin R, Melotti R, Barin L et al. Cohort profile: the cooperative health research in south tyrol study. Int J Epidemiol. 2025;54:dyaf064. 10.1093/ije/dyaf064 40436620 PMC12119133

[17] Rebholz CM, Crews DC, Grams ME et al. DASH (Dietary Approaches to Stop Hypertension) diet and risk of subsequent kidney disease. Am J Kidney Dis. 2016;68:853–61. 10.1053/j.ajkd.2016.05.019 27519166 PMC5123940

[18] Chiuve SE, Fung TT, Rimm EB et al. Alternative dietary indices both strongly predict risk of chronic disease. J Nutr. 2012;142:1009–18. 10.3945/jn.111.157222 22513989 PMC3738221

[19] Trichopoulou A, Costacou T, Bamia C et al. Adherence to a Mediterranean diet and survival in a Greek population. N Engl J Med. 2003;348:2599–608. 10.1056/NEJMoa025039 12826634

[20] Wuttke M, Li Y, Li M et al. A catalog of genetic loci associated with kidney function from analyses of a million individuals. Nat Genet. 2019;51:957–72. 10.1038/s41588-019-0407-x 31152163 PMC6698888

[21] Yu Z, Coresh J, Qi G et al. A bidirectional Mendelian randomization study supports causal effects of kidney function on blood pressure. Kidney Int. 2020;98:708–16. 10.1016/j.kint.2020.04.044 32454124 PMC7784392

[22] Pattaro C, Gögele M, Mascalzoni D et al. The Cooperative Health Research in South Tyrol (CHRIS) study: rationale, objectives, and preliminary results. J Transl Med. 2015;13:348. 10.1186/s12967-015-0704-9 26541195 PMC4635524

[23] Noce D, Gögele M, Schwienbacher C et al. Sequential recruitment of study participants may inflate genetic heritability estimates. Hum Genet. 2017;136:743–57. 10.1007/s00439-017-1785-8 28374192

[24] Inker LA, Eneanya ND, Coresh J et al. New creatinine- and cystatin C–Based equations to estimate GFR without race. N Engl J Med. 2021;385:1737–49. 10.1056/NEJMoa2102953 34554658 PMC8822996

[25] Pattaro C, Riegler P, Stifter G et al. Estimating the glomerular filtration rate in the general population using different equations: effects on classification and association. Nephron Clin Pract. 2013;123:102–11. 10.1159/000351043 23797027

[26] Barbieri G, Cazzoletti L, Melotti R et al. Development and evaluation of a kidney health questionnaire and estimates of chronic kidney disease prevalence in the Cooperative Health Research in South Tyrol (CHRIS) study. J Nephrol Published online November 27, 2024; 38:521–30. 10.1007/s40620-024-02157-6 39602026 PMC11961529

[27] Garcia-Larsen V, Luczynska M, Kowalski ML et al. Use of a common food frequency questionnaire (FFQ) to assess dietary patterns and their relation to allergy and asthma in Europe: pilot study of the GA2LEN FFQ. Eur J Clin Nutr. 2011;65:750–6. 10.1038/ejcn.2011.15 21427744

[28] McCance and Widdowson’s The Composition of Foods Integrated Dataset 2021. 2021.

[29] Picard K, Barreto Silva MI, Mager D et al. Dietary potassium intake and risk of chronic kidney disease progression in predialysis patients with chronic kidney disease: a systematic review. Adv Nutr. 2020;11:1002–15. 10.1093/advances/nmaa027 32191264 PMC7360460

[30] Palmer SC . Serum levels of phosphorus, parathyroid hormone, and calcium and risks of death and cardiovascular disease in individuals with chronic kidney disease: a systematic review and meta-analysis. JAMA. 2011;305:1119–27. 10.1001/jama.2011.308 21406649

[31] Barbieri G, Garcia-Larsen V, Lundin R et al. Associations between dietary patterns and kidney health assessed in the population-based CHRIS study using reduced rank regression. J Ren Nutr Published online March 21, 2024; 34:427–37. S1051-2276(24)00051-7. 10.1053/j.jrn.2024.03.003 38521380

[32] Fung TT . Adherence to a DASH-style diet and risk of coronary heart disease and stroke in women. Arch Intern Med. 2008;168:713–20. 10.1001/archinte.168.7.713 18413553

[33] Willett WC, Howe GR, Kushi LH. Adjustment for total energy intake in epidemiologic studies. Am J Clin Nutr. 1997;65:1220S–8S. 10.1093/ajcn/65.4.1220S 9094926

[34] Ghasemi-Semeskandeh D, Emmert D, König E et al. Systematic mediation and interaction analyses of kidney function genetic loci in a general population study. PLoS One. 2025;20:e0323057. 10.1371/journal.pone.0323057 40554574 PMC12186986

[35] Michels KB, Willett WC. Self-administered semiquantitative food frequency questionnaires: patterns, predictors, and interpretation of omitted items. Epidemiology. 2009;20:295–301. 10.1097/EDE.0b013e3181931515 19106799 PMC3935217

[36] Buuren S, Groothuis-Oudshoorn K. Mice: multivariate imputation by chained equations in R. J Stat Softw. 2011;45:1–67. 10.18637/jss.v045.i03

[37] Craig CL, Marshall AL, Sjöström M et al. International physical activity questionnaire: 12-country reliability and validity. Med Sci Sports Exerc. 2003;35:1381–95. 10.1249/01.MSS.0000078924.61453.FB 12900694

[38] Murgia F, Melotti R, Foco L et al. Effects of smoking status, history and intensity on heart rate variability in the general population: the CHRIS study. PLoS One. 2019;14:e0215053. 10.1371/journal.pone.0215053 30964923 PMC6456196

[39] Ge T, Chen CY, Ni Y et al. Polygenic prediction via bayesian regression and continuous shrinkage priors. Nat Commun. 2019;10:1776. 10.1038/s41467-019-09718-5 30992449 PMC6467998

[40] Kidney Disease: Improving Global Outcomes (KDIGO) CKD-MBD Work Group . KDIGO clinical practice guideline for the diagnosis, evaluation, prevention, and treatment of Chronic Kidney Disease-mineral and Bone disorder (CKD-MBD). Kidney Int Suppl. 2009;:S1–130. 10.1038/ki.2009.188 19644521

[41] Musgrove J, Wolf M Regulation and effects of FGF23 in chronic kidney disease. Annu Rev Physiol. 2020;82:365–90. 10.1146/annurev-physiol-021119-034650 31743079

[42] Juraschek SP, Appel LJ, Anderson CAM et al. Effect of a high-protein diet on kidney function in healthy adults: results from the OmniHeart trial. Am J Kidney Dis. 2013;61:547–54. 10.1053/j.ajkd.2012.10.017 23219108 PMC3602135

[43] Cirillo M, Laurenzi M, Mancini M et al. Low glomerular filtration in the population: prevalence, associated disorders, and awareness. Kidney Int. 2006;70:800–6. 10.1038/sj.ki.5001641 16820784

[44] Lee BY, Ordovás JM, Parks EJ et al. Research gaps and opportunities in precision nutrition: an NIH workshop report. Am J Clin Nutr. 2022;116:1877. 10.1093/ajcn/nqac237 36055772 PMC9761773

[45] Guasch-Ferré M, Wittenbecher C, Palmnäs M et al. Precision nutrition for cardiometabolic diseases. Nat Med. 2025;31:1444–53. 10.1038/s41591-025-03669-9 40307513

[46] EFSA Panel on Dietetic Products, Nutrition and Allergies (NDA) . Scientific opinion on dietary reference values for protein. EFSA J. 2012;10:2557. 10.2903/j.efsa.2012.2557

[47] Smyth A, Griffin M, Yusuf S et al. Diet and major renal outcomes: a prospective cohort study. The NIH-AARP Diet and Health study. J Ren Nutr. 2016;26:288–98. 10.1053/j.jrn.2016.01.016 26975776

[48] Missikpode C, Ricardo AC, Durazo-Arvizu RA et al. Association of diet quality indices with longitudinal changes in kidney function in U.S. Hispanics/Latinos: findings from the Hispanic Community Health Study/Study of Latinos (HCHS/SOL). Kidney360. 2020;2:50–62. 10.34067/KID.0004552020 35368818 PMC8785733

[49] Hu EA, Steffen LM, Grams ME et al. Dietary patterns and risk of incident chronic kidney disease: the Atherosclerosis Risk in Communities study. Am J Clin Nutr. 2019;110:713–21. 10.1093/ajcn/nqz146 31386145 PMC6736122

[50] Willett W . Nutritional Epidemiology. Vol 40. Oxford Press; 2012. 10.1093/acprof:oso/9780199754038.001.0001

[51] Rothman KJ, Greenland S, Lash TL. Modern Epidemiology. 3rd ed. Wolters Kluwer health—Lippincott Williams & Wilkins; 2008;.

[52] Levey AS, Inker LA, Coresh J GFR estimation: from physiology to public health. Am J Kidney Dis. 2014;63:820–34. 10.1053/j.ajkd.2013.12.006 24485147 PMC4001724

[53] Pattaro C, Teumer A, Gorski M et al. Genetic associations at 53 loci highlight cell types and biological pathways relevant for kidney function. Nat Commun. 2016;7:10023. 10.1038/ncomms10023 26831199 PMC4735748

[54] Hussain J, Grubic N, Akbari A et al. Associations between modest reductions in kidney function and adverse outcomes in young adults: retrospective, population based cohort study. BMJ. 2023;381:e075062. 10.1136/bmj-2023-075062 37353230 PMC10286512

[55] Nair S, O’Brien SV, Hayden K et al. Effect of a cooked meat meal on serum creatinine and estimated glomerular filtration rate in diabetes-related kidney disease. Diabetes Care. 2014;37:483–7. 10.2337/dc13-1770 24062331

