# Supplementary material for: Combined effects of diet and genetic predisposition on kidney function outcomes
Source: Clin Kidney J. 2026 Jun 19;19(8):sfag196. doi: 10.1093/ckj/sfag196 (PMC13492336; doi:10.1093/ckj/sfag196)
Supplement: Supplementary file 1 [file sfag196_supplemental_file.docx]

**Figure S1**: Flowchart of the study sample selection.

**Figure S2**: Correlation Heatmap and PGS Distribution. a) Histogram showing the relative distribution (%) of PGS values within the study population. b) Heatmap of Pearson correlations among exposure variables, including dietary exposures* and the polygenic score (PGS).

*Abbreviations*: PGS, polygenic score; eGFR, estimated glomerular filtration rate.

*** *Dietary exposure*: Proteins, Potassium, Phosphorus, AHEI, Mediterranean, DASH

**Figure S3a**: Heatmaps showing predicted eGFRcrea levels across combinations of dietary exposures* and PGS, derived from the minimally adjusted** GAM with non-linear tensor product smooths. The color gradient represents predicted eGFRcrea levels, with red colors indicating lower levels and green colors indicating higher eGFRcrea levels.

s

*** *Dietary exposure*: Proteins, Potassium, Phosphorus, AHEI, Mediterranean, DASH

***Minimally adjusted*: Adjusted for age, sex and the first 10 genetic principal components (PCs)

*Abbreviations:* GAM, generalized additive model; PGS, polygenic score; eGFR, estimated glomerular filtration rate.

**Figure S3b:** Predicted marginal effects of dietary exposures* on eGFRcrea for individuals in the lowest (1st tertile) and highest (3rd tertile) PGS groups, derived from the minimally adjusted** GAM with non-linear tensor product smooths. Solid lines represent predicted eGFRcrea, and shaded ribbons indicate 95% confidence intervals.

*** *Dietary exposure*: Proteins, Potassium, Phosphorus, AHEI, Mediterranean, DASH

***Minimally adjusted*: Adjusted for age, sex and the first 10 genetic principal components (PCs)

*Abbreviations:* GAM, generalized additive model; PGS, polygenic score; eGFR, estimated glomerular filtration rate.

**Figure S4a**: Heatmaps showing predicted eGFRcrea levels across combinations of dietary exposures* and PGS, derived from the fully adjusted** GAM with non-linear tensor product smooths. The color gradient represents predicted eGFRcrea levels, with red colors indicating lower levels and green colors indicating higher eGFRcrea levels.

*** *Dietary exposure*: Proteins, Potassium, Phosphorus, AHEI, Mediterranean, DASH

***Fully adjusted*: Adjusted for age, sex, BMI, physical activity, smoking habits, education and the first 10 genetic principal components (PCs)

*Abbreviations:* GAM, generalized additive model; PGS, polygenic score; eGFR, estimated glomerular filtration rate.

**Figure S4b:** Predicted marginal effects of dietary exposures* on eGFRcrea for individuals in the lowest (1st tertile) and highest (3rd tertile) PGS groups, derived from the minimally adjusted** GAM with non-linear tensor product smooths. Solid lines represent predicted eGFRcrea, and shaded ribbons indicate 95% confidence intervals.

*** *Dietary exposure*: Proteins, Potassium, Phosphorus, AHEI, Mediterranean, DASH

***Fully adjusted*: Adjusted for age, sex, BMI, physical activity, smoking habits, education and the first 10 genetic principal components (PCs)

*Abbreviations:* GAM, generalized additive model; PGS, polygenic score; eGFR, estimated glomerular filtration rate.

**Figure S5**: Scatterplot comparing the two polygenic scores (PGS): the primary score and *PGS_eGFRcrea_*. Each point represents an individual’s values for both scores (Pearson’s correlation coefficient = 0.45).

**Table S1**: ATC codes used to identify self-reported medications for hypertension and diabetes

| Type of medication | Therapy | ATC code |
| --- | --- | --- |
| Blood pressure lowering drugs | Antihypertensives | C02AC01, C02CA04, C02LA01 |
|  | Diuretics | C03AA03, C03BA08, C03BA11, C03CA01, C03CA04, C03DA01, C03DA02, C03DA04, C03EA01, C03EB01 |
|  | Peripheral vasodilators | C04AD03, C04AE01, C04AE51 |
|  | Beta blocking agents | C07, C07AA05, C07AA06, C07AA07, C07AB02, C07AB03, C07AB07, C07AB12, C07AG02, C07B, C07BB12, C07CB03 |
|  | Calcium channel blockers | C08CA01, C08CA02, C08CA05, C08CA09, C08CA11, C08CA12, C08CA13, C08DA01, C08DB01 |
|  | Agents acting on the renin-angiotensin system | C09AA02, C09AA03, C09AA04, C09AA05, C09AA06, C09AA07, C09AA09, C09AA15, C09BA01, C09BA02, C09BA03, C09BA04, C09BA05, C09BA06, C09BA09, C09BA12, C09BA15, C09BB02, C09BB04, C09BB07, C09BX01, C09CA01, C09CA03, C09CA04, C09CA06, C09CA07, C09CA08, C09DA, C09DA01, C09DA03, C09DA04, C09DA06, C09DA07, C09DA08, C09DB02 |
| Drugs used in diabetes | Insulins and analogues | A10AB04, A10AB05, A10AB06, A10AD05, A10AE04, A10AE05, A10AE06 |
|  | Blood glucose lowering drugs (excl. insulins) | A10BA02, A10BB09, A10BB12, A10BD02, A10BD05, A10BD06, A10BD07, A10BD08, A10BD13, A10BG03, A10BH01, A10BH03, A10BK01, A10BX02, A10BX07 |

**Table S2**: Variants included in the computation of PGS with imputation score and allele frequency in the CHRIS dataset.

| **RS number** | **CHR** | **BP** | **NEA** | **EA** | **Rsq Imputation Score** |  | **EAF** | **Locus name*** |
| --- | --- | --- | --- | --- | --- | --- | --- | --- |
| rs10159261 | 1 | 15912987 | G | T | 0.99147 |  | 0.28 | AGMAT |
| rs17413465 | 1 | 55718708 | C | A | 0.98907 |  | 0.21 | MIR4422 |
| rs1757915 | 1 | 56615809 | G | A | 0.99989 |  | 0.36 | PPAP2B |
| rs679843 | 1 | 78707493 | C | T | 0.96461 |  | 0.24 | MGC27382 |
| rs1887252 | 1 | 82957871 | G | C | 0.97248 |  | 0.35 | LOC101927498 |
| rs7543734 | 1 | 94050911 | G | C | 0.97815 |  | 0.19 | BCAR3 |
| rs11166440 | 1 | 1.01E+08 | A | G | 0.99894 |  | 0.38 | CDC14A |
| rs267738 | 1 | 1.51E+08 | T | G | 0.99987 |  | 0.17 | CERS2 |
| rs4971100 | 1 | 1.55E+08 | G | A | 0.98559 |  | 0.39 | TRIM46 |
| rs1011731 | 1 | 1.72E+08 | G | A | 0.99987 |  | 0.39 | DNM3 |
| rs3795503 | 1 | 1.81E+08 | C | T | 0.93734 |  | 0.32 | KIAA1614 |
| rs3850625 | 1 | 2.01E+08 | G | A | 0.99427 |  | 0.12 | CACNA1S |
| rs2808454 | 1 | 2.07E+08 | A | T | 0.9774 |  | 0.41 | PFKFB2 |
| rs2577134 | 1 | 2.2E+08 | C | T | 0.95776 |  | 0.24 | RNU5F-1 |
| rs2490391 | 1 | 2.43E+08 | A | C | 0.94622 |  | 0.49 | SDCCAG8 |
| rs3791221 | 2 | 226933 | A | G | 1 |  | 0.11 | SH3YL1 |
| rs807624 | 2 | 15782471 | G | T | 0.99994 |  | 0.46 | DDX1 |
| rs6546869 | 2 | 73895765 | G | A | 0.98855 |  | 0.16 | ALMS1P |
| rs11123169 | 2 | 1.14E+08 | C | T | 0.99585 |  | 0.31 | PSD4 |
| rs17050272 | 2 | 1.21E+08 | G | A | 0.99072 |  | 0.43 | LINC01101 |
| rs11694902 | 2 | 1.22E+08 | G | A | 0.98185 |  | 0.07 | TFCP2L1 |
| rs7425436 | 2 | 1.49E+08 | A | G | 0.98556 |  | 0.46 | ORC4 |
| rs35284526 | 2 | 1.78E+08 | C | A | 0.98226 |  | 0.45 | NFE2L2 |
| rs4666821 | 2 | 1.83E+08 | G | T | 0.98363 |  | 0.15 | PDE1A |
| rs7651407 | 3 | 48443816 | C | T | 0.9718 |  | 0.2 | PLXNB1 |
| rs3774726 | 3 | 63974477 | C | T | 0.98451 |  | 0.43 | ATXN7 |
| rs2289746 | 3 | 1.05E+08 | T | C | 0.99983 |  | 0.2 | CBLB |
| rs9868185 | 3 | 1.22E+08 | A | G | 0.99322 |  | 0.5 | SLC15A2 |
| rs35320690 | 3 | 1.36E+08 | T | C | 0.98725 |  | 0.22 | MSL2 |
| rs9828976 | 3 | 1.37E+08 | C | G | 0.98726 |  | 0.33 | SLC35G2 |
| rs76272256 | 3 | 1.69E+08 | C | T | 0.98513 |  | 0.35 | MECOM |
| rs56065557 | 3 | 1.85E+08 | G | C | 0.98876 |  | 0.18 | SENP2 |
| rs11919484 | 3 | 1.86E+08 | G | T | 0.98572 |  | 0.27 | KNG1 |
| rs9823161 | 3 | 1.94E+08 | A | G | 0.94684 |  | 0.45 | HES1 |
| rs3775932 | 4 | 10090930 | C | A | 0.98335 |  | 0.44 | WDR1 |
| rs28817415 | 4 | 77401452 | C | T | 0.99251 |  | 0.08 | SHROOM3 |
| rs12509595 | 4 | 81182554 | T | C | 0.98298 |  | 0.18 | FGF5 |
| rs223471 | 4 | 1.04E+08 | G | C | 0.98617 |  | 0.18 | MANBA |
| rs13157326 | 5 | 34504277 | G | A | 0.96608 |  | 0.29 | RAI14 |
| rs1362800 | 5 | 39378115 | C | T | 0.97987 |  | 0.4 | DAB2 |
| rs11746506 | 5 | 44812566 | C | T | 1 |  | 0.14 | MRPS30 |
| rs12520984 | 5 | 52787358 | G | C | 0.98822 |  | 0.24 | FST |
| rs79760705 | 5 | 53298716 | G | T | 0.99318 |  | 0.47 | ARL15 |
| rs72759880 | 5 | 67750213 | G | T | 0.99406 |  | 0.09 | PIK3R1 |
| rs2010352 | 5 | 68656327 | G | A | 0.96813 |  | 0.34 | AK6 |
| rs12777 | 5 | 1.32E+08 | C | G | 0.97663 |  | 0.19 | SLC22A4 |
| rs12163971 | 5 | 1.32E+08 | C | A | 0.98881 |  | 0.26 | AFF4 |
| rs11743174 | 5 | 1.49E+08 | C | T | 0.94549 |  | 0.48 | ABLIM3 |
| rs3812036 | 5 | 1.77E+08 | C | T | 0.93847 |  | 0.07 | SLC34A1 |
| rs11755724 | 6 | 7118990 | A | G | 0.99763 |  | 0.33 | RREB1 |
| rs3765502 | 6 | 24354045 | T | C | 0.99938 |  | 0.18 | DCDC2 |
| rs13200335 | 6 | 41690823 | C | A | 0.98013 |  | 0.24 | TFEB |
| rs77915916 | 6 | 43287722 | A | T | 0.97973 |  | 0.27 | CRIP3 |
| rs720989 | 6 | 44765535 | T | G | 0.99647 |  | 0.16 | SUPT3H |
| rs6458868 | 6 | 52630153 | C | T | 0.95384 |  | 0.14 | GSTA2 |
| rs3925003 | 6 | 55422618 | C | T | 0.99865 |  | 0.06 | HMGCLL1 |
| rs72912510 | 6 | 90118764 | G | A | 0.97045 |  | 0.38 | RRAGD |
| rs1857859 | 6 | 1.01E+08 | G | A | 0.99988 |  | 0.15 | SIM1 |
| rs1268168 | 6 | 1.09E+08 | G | A | 0.98576 |  | 0.31 | FOXO3 |
| rs7740107 | 6 | 1.3E+08 | T | A | 0.95405 |  | 0.25 | L3MBTL3 |
| rs3822939 | 6 | 1.34E+08 | A | G | 0.98645 |  | 0.1 | EYA4 |
| rs9397738 | 6 | 1.55E+08 | G | A | 0.99831 |  | 0.32 | SCAF8 |
| rs6968554 | 7 | 17287106 | A | G | 0.99807 |  | 0.27 | AHR |
| rs700753 | 7 | 46753684 | C | G | 0.97858 |  | 0.32 | TNS3 |
| rs55773927 | 7 | 65337902 | T | C | 0.93184 |  | 0.34 | VKORC1L1 |
| rs801193 | 7 | 66030612 | T | G | 0.96145 |  | 0.3 | LOC493754 |
| rs41301394 | 7 | 75612803 | C | T | 0.98769 |  | 0.22 | POR |
| rs6973656 | 7 | 77422583 | A | G | 0.98369 |  | 0.34 | TMEM60 |
| rs62491533 | 7 | 1.3E+08 | T | C | 0.98583 |  | 0.44 | UBE2H |
| rs34861762 | 8 | 23748420 | C | T | 0.98524 |  | 0.14 | STC1 |
| rs2039424 | 9 | 71432174 | G | A | 0.97774 |  | 0.34 | PIP5K1B |
| rs1321917 | 9 | 1.19E+08 | G | C | 0.98184 |  | 0.38 | ASTN2 |
| rs80282103 | 10 | 899071 | A | T | 0.98359 |  | 0.5 | LARP4B |
| rs7072591 | 10 | 35150364 | A | G | 0.96529 |  | 0.06 | PARD3-AS1 |
| rs10821905 | 10 | 52646093 | G | A | 0.99993 |  | 0.19 | A1CF |
| rs10821944 | 10 | 63785089 | G | T | 0.95822 |  | 0.19 | ARID5B |
| rs7475348 | 10 | 69965177 | C | T | 0.953 |  | 0.36 | MYPN |
| rs12240572 | 10 | 75016365 | T | A | 0.96609 |  | 0.43 | CFAP70 |
| rs7095954 | 10 | 82209232 | A | T | 0.98306 |  | 0.35 | TSPAN14 |
| rs2068888 | 10 | 94839642 | G | A | 0.99632 |  | 0.19 | CYP26A1 |
| rs4918943 | 10 | 97278922 | G | A | 0.99414 |  | 0.2 | SORBS1 |
| rs284859 | 10 | 1.05E+08 | G | T | 0.98855 |  | 0.46 | WBP1L |
| rs1055256 | 10 | 1.26E+08 | A | G | 0.99997 |  | 0.32 | METTL10 |
| rs11564722 | 11 | 2178330 | C | T | 0.93638 |  | 0.46 | INS-IGF2 |
| rs963837 | 11 | 30749090 | T | C | 0.9173 |  | 0.31 | DCDC5 |
| rs6484504 | 11 | 31424823 | T | C | 0.99997 |  | 0.33 | DNAJC24 |
| rs7127946 | 11 | 48250675 | C | T | 0.95189 |  | 0.44 | OR4B1 |
| rs3892895 | 11 | 68884755 | A | G | 0.99889 |  | 0.27 | TPCN2 |
| rs11237450 | 11 | 78023356 | C | A | 0.95676 |  | 0.25 | GAB2 |
| rs10790452 | 11 | 1.22E+08 | C | T | 0.95591 |  | 0.14 | SORL1 |
| rs11062167 | 12 | 364739 | G | A | 0.97289 |  | 0.29 | SLC6A13 |
| rs632887 | 12 | 3392351 | A | G | 0.99786 |  | 0.31 | TSPAN9 |
| rs117113238 | 12 | 12209203 | G | A | 0.98863 |  | 0.36 | BCL2L14 |
| rs10846157 | 12 | 15325031 | A | C | 0.9816 |  | 0.48 | RERG |
| rs12313306 | 12 | 57751854 | C | T | 0.98664 |  | 0.44 | R3HDM2 |
| rs1275609 | 12 | 76271183 | G | A | 0.9871 |  | 0.29 | PHLDA1 |
| rs500830 | 13 | 72348768 | C | T | 0.97331 |  | 0.34 | DACH1 |
| rs17184313 | 14 | 93102251 | C | T | 0.99997 |  | 0.46 | RIN3 |
| rs690428 | 15 | 53950578 | A | C | 0.98914 |  | 0.42 | WDR72 |
| rs11071738 | 15 | 63580155 | T | C | 0.98019 |  | 0.35 | APH1B |
| rs11071939 | 15 | 67463391 | T | C | 0.9846 |  | 0.36 | SMAD3 |
| rs351237 | 15 | 74477239 | G | A | 0.92444 |  | 0.11 | STRA6 |
| rs2472297 | 15 | 75027880 | C | T | 0.99414 |  | 0.09 | CYP1A1 |
| rs4886696 | 15 | 75664570 | A | T | 0.9521 |  | 0.45 | SIN3A |
| rs4886755 | 15 | 76298132 | A | G | 0.97132 |  | 0.05 | NRG4 |
| rs166906 | 15 | 76802175 | C | T | 0.99578 |  | 0.16 | SCAPER |
| rs59646751 | 15 | 99276521 | G | T | 0.96552 |  | 0.26 | IGF1R |
| rs77924615 | 16 | 20392332 | G | A | 0.92796 |  | 0.24 | PDILT |
| rs9932625 | 16 | 51735746 | G | A | 0.99099 |  | 0.38 | LOC101927364 |
| rs7203398 | 16 | 53189672 | A | C | 0.99431 |  | 0.08 | CHD9 |
| rs62050038 | 16 | 69802865 | A | T | 0.97761 |  | 0.4 | WWP2 |
| rs28581385 | 16 | 79942679 | A | T | 0.99374 |  | 0.09 | LOC101928248 |
| rs28735420 | 17 | 12139964 | T | G | 0.95676 |  | 0.22 | MAP2K4 |
| rs2411192 | 17 | 34882998 | A | T | 0.95682 |  | 0.34 | MYO19 |
| rs9903801 | 17 | 58915261 | G | C | 0.9708 |  | 0.41 | BCAS3 |
| rs8866 | 17 | 65373979 | C | G | 0.97438 |  | 0.22 | PITPNC1 |
| rs883541 | 17 | 66449122 | G | A | 0.99992 |  | 0.28 | PRKAR1A,WIPI1 |
| rs16942751 | 18 | 24393213 | C | A | 0.9669 |  | 0.43 | AQP4 |
| rs7251730 | 19 | 36997147 | C | T | 0.98976 |  | 0.25 | ZNF260 |
| rs78241494 | 19 | 37649748 | T | C | 0.99037 |  | 0.45 | ZNF585A |
| rs113445505 | 19 | 38157969 | C | T | 0.9889 |  | 0.16 | ZNF781 |
| rs62187537 | 20 | 1333060 | C | T | 0.98399 |  | 0.4 | FKBP1A-SDCBP2 |
| rs17216707 | 20 | 52732362 | T | C | 0.94679 |  | 0.36 | CYP24A1 |
| rs2235826 | 20 | 56143169 | T | A | 0.96188 |  | 0.43 | PCK1 |
| rs1407040 | 20 | 57472174 | C | T | 0.96945 |  | 0.44 | GNAS |
| rs4408777 | 20 | 62706105 | A | G | 0.96345 |  | 0.25 | RGS19 |
| rs2823139 | 21 | 16576783 | G | A | 0.99898 |  | 0.43 | NRIP1 |
| rs2834317 | 21 | 35356706 | G | A | 0.97924 |  | 0.16 | LINC00649 |
| rs2244237 | 21 | 37818141 | G | T | 0.99118 |  | 0.41 | CLDN14 |
| rs4820324 | 22 | 38599857 | G | C | 0.9671 |  | 0.41 | MAFF |
| rs738527 | 22 | 43112961 | T | C | 0.99946 |  | 0.49 | A4GALT |

*According to Kidney GenePrioritiSation – KidneyGPS tool at <https://kidneygps.ur.de/gps/> (Stanzick et al, Nat Commun 2021) and UCSC Genome Browser (<https://genome.ucsc.edu>).

**Table S3.** Variance inflation factors (VIFs) for all parametric covariates included in the fully adjusted models; VIF values close to 1 indicate negligible multicollinearity, whereas values above 5 may indicate potential concern.

| VIF | **Dietary exposure** | | | | | |
| --- | --- | --- | --- | --- | --- | --- |
|  | **Proteins** | **Potassium** | **Phosphorus** | **AHEI** | **Med score** | **DASH** |
| Sex - F | 1.23 | 1.12 | 1.13 | 1.19 | 1.12 | 1.12 |
| Physical activity | 1.06 | 1.06 | 1.06 | 1.06 | 1.06 | 1.06 |
| Smoke - Current smoker | 1.14 | 1.13 | 1.14 | 1.15 | 1.14 | 1.15 |
| Smoke - Past smoker | 1.15 | 1.15 | 1.15 | 1.15 | 1.15 | 1.15 |
| BMI | 1.14 | 1.13 | 1.13 | 1.14 | 1.13 | 1.13 |
| Education - Lower secondary school | 4.12 | 4.13 | 4.13 | 4.12 | 4.12 | 4.12 |
| Education - Upper secondary school | 6.44 | 6.48 | 6.47 | 6.53 | 6.50 | 6.50 |
| Education - Vocational school | 7.04 | 7.08 | 7.07 | 7.09 | 7.08 | 7.07 |
| Education - University or higher | 3.72 | 3.75 | 3.74 | 3.79 | 3.77 | 3.77 |
| Total Energy Intake | 1.03 | 1.03 | 1.03 | 1.03 | 1.05 | 1.04 |
| PC1 | 1.19 | 1.18 | 1.18 | 1.18 | 1.18 | 1.18 |
| PC2 | 1.10 | 1.10 | 1.10 | 1.10 | 1.10 | 1.10 |
| PC3 | 1.34 | 1.34 | 1.34 | 1.34 | 1.34 | 1.34 |
| PC4 | 1.07 | 1.07 | 1.07 | 1.07 | 1.07 | 1.07 |
| PC5 | 1.16 | 1.16 | 1.16 | 1.16 | 1.16 | 1.16 |
| PC6 | 1.02 | 1.02 | 1.02 | 1.02 | 1.02 | 1.02 |
| PC7 | 1.12 | 1.12 | 1.12 | 1.12 | 1.12 | 1.12 |
| PC8 | 1.18 | 1.18 | 1.18 | 1.18 | 1.17 | 1.18 |
| PC9 | 1.03 | 1.03 | 1.03 | 1.03 | 1.03 | 1.03 |
| PC10 | 1.06 | 1.06 | 1.06 | 1.06 | 1.06 | 1.06 |

**Table S4**: Model diagnostics for the GAM analysis on the association of PGS and dietary exposures with eGFRcrea, including overfitting checks and Akaike Information Criterion (AIC) values.

|  | Minimally adjusted model* | | | | Fully adjusted model** | | | |
| --- | --- | --- | --- | --- | --- | --- | --- | --- |
| **Main effects models^#^** | df | Ref. df | AIC | Total df | df | Ref. df | AIC | Total df |
| Proteins | 1.00 | 1.01 | 42965.02 | 3.38 | 1.00 | 1.01 | 42913.96 | 3.23 |
| Age | 2.38 | 2.99 |  |  | 2.22 | 2.82 |  |  |
| Potassium | 1.00 | 1.00 | 42973.2 | 3.43 | 1.00 | 1.00 | 42925.07 | 3.29 |
| Age | 2.43 | 3.06 |  |  | 2.29 | 2.91 |  |  |
| Phosphorus | 1.00 | 1.00 | 42978.68 | 3.39 | 1.00 | 1.01 | 42929.44 | 3.25 |
| Age | 2.39 | 3.01 |  |  | 2.24 | 2.85 |  |  |
| AHEI | 1.63 | 2.05 | 42980.09 | 4.07 | 1.00 | 1.01 | 42931.14 | 3.26 |
| Age | 2.44 | 3.07 |  |  | 2.26 | 2.86 |  |  |
| Mediterranean | 1.00 | 1.00 | 42982.61 | 3.44 | 1.00 | 1.00 | 42930.83 | 3.24 |
| Age | 2.44 | 3.07 |  |  | 2.24 | 2.84 |  |  |
| DASH | 2.11 | 2.67 | 42978.52 | 4.51 | 1.94 | 2.46 | 42926.08 | 4.16 |
| Age | 2.41 | 3.03 |  |  | 2.23 | 2.83 |  |  |
| **Interaction models^##^** | df | Ref. df | AIC | Total df | df | Ref. df | AIC | Total df |
| Proteins:PGS | 4.91 | 6.55 | 42733.22 | 9.30 | 4.81 | 6.44 | 42686.41 | 8.99 |
| Proteins | 1.00 | 1.00 |  |  | 1.00 | 1.01 |  |  |
| PGS | 1.00 | 1.01 |  |  | 1.00 | 1.00 |  |  |
| Age | 2.39 | 3.01 |  |  | 2.17 | 2.76 |  |  |
| Potassium:PGS | 1.00 | 1.01 | 42750.34 | 5.45 | 1.01 | 1.01 | 42706.52 | 5.27 |
| Potassium | 1.00 | 1.00 |  |  | 1.00 | 1.00 |  |  |
| PGS | 1.00 | 1.00 |  |  | 1.00 | 1.01 |  |  |
| Age | 2.44 | 3.07 |  |  | 2.25 | 2.86 |  |  |
| Phosphorus:PGS | 5.40 | 7.13 | 42747.05 | 9.84 | 5.49 | 7.23 | 42702.00 | 9.73 |
| Phosphorus | 1.00 | 1.00 |  |  | 1.00 | 1.00 |  |  |
| PGS | 1.01 | 1.01 |  |  | 1.01 | 1.01 |  |  |
| Age | 2.44 | 3.07 |  |  | 2.24 | 2.84 |  |  |
| AHEI:PGS | 1.05 | 1.09 | 42755.79 | 6.67 | 1.04 | 1.07 | 42707.02 | 6.34 |
| AHEI | 1.94 | 2.41 |  |  | 1.57 | 1.95 |  |  |
| PGS | 1.00 | 1.01 |  |  | 1.00 | 1.01 |  |  |
| Age | 2.45 | 3.08 |  |  | 2.20 | 2.79 |  |  |
| Mediterranean:PGS | 1.00 | 1.00 | 42757.79 | 6.43 | 1.01 | 1.02 | 42712.52 | 5.81 |
| Mediterranean | 1.02 | 1.03 |  |  | 1.02 | 1.04 |  |  |
| PGS | 1.00 | 1.00 |  |  | 1.00 | 1.01 |  |  |
| Age | 2.45 | 3.08 |  |  | 2.20 | 2.79 |  |  |
| DASH:PGS | 1.02 | 1.03 | 42760.41 | 5.47 | 1.02 | 1.03 | 42712.10 | 5.23 |
| DASH | 2.24 | 2.75 |  |  | 2.16 | 2.65 |  |  |
| PGS | 1.00 | 1.01 |  |  | 1.00 | 1.01 |  |  |
| Age | 2.40 | 3.03 |  |  | 2.15 | 2.73 |  |  |

*Abbreviations*: GAM, generalized additive model; PGS, polygenic score; eGFR, estimated glomerular filtration rate; df, degrees of freedom; Ref. df, reference degrees of freedom; Total df, total degrees of freedom; AIC, Akaike Information Criterion.

*Adjusted for: age, sex, and the first 10 genetic principal components (PCs) of the genotyped data

**Adjusted for: age, sex, first 10 genetic PCs of the genotyped data, body mass index, physical activity, smoking habits, and education.

** *Dietary exposure*: Proteins, Potassium, Phosphorus, AHEI, Mediterranean, DASH

^#^ Each term was included in a separate, minimally adjusted and fully adjusted models

^##^ Main effects and their interaction were included in the minimally adjusted and fully adjusted models

**Table S5**: Proportion of variance in eGFRcrea explained by dietary and genetic exposure variables, estimated using unadjusted* linear models. Explained variance is calculated using the adjusted $R^{2}$statistic for each model.

|  | Variable | R^2^ |
| --- | --- | --- |
| PGS | PGS | 1.46 |
|  | PRS-CS | 5.68 |
| Dietary esposures | Proteins | 1.75 |
|  | Potassium | 1.92 |
|  | Phosphorus | 0.22 |
|  | AHEI-score | 1.87 |
|  | Med-score | 0.39 |
|  | DASH-score | 2.23 |

*Abbreviations:* PGS, polygenic score; eGFR, estimated glomerular filtration rate.

* Unadjusted models:

PGS model: $eGFRcrea=\beta0+\beta1\times PGS$

Dietary exposure model: $eGFRcrea=\beta0+{\beta1\times Dietary exposure}^{**}$

** Dietary exposure: Proteins, Potassium, Phosphorus, AHEI, Mediterranean, DASH

**Table S6**: Results of linear regression analysis on the association between the exposure variables (dietary and genetics), and eGFRcrea. The table reports coefficients, 95% confidence intervals (95%CIs), and p-values for the main effects models for PGS ($eGFRcrea=\beta0+\beta1\times PGS+\mathrm{adjustments}^{*}$), and dietary exposure** ($eGFRcrea=\beta0+\beta1\times{dietary exposure}^{**}+\mathrm{adjustments}^{*}$).

|  | Minimally adjusted* | |  | Fully adjusted* | |
| --- | --- | --- | --- | --- | --- |
|  | Coefficient (95% CI) | p-value |  | Coefficient (95% CI) | p-value |
| PGS | -1.7239 (-1.9874, -1.4604) | <0.00001 |  | -1.7007 (-1.963, -1.4384) | <0.00001 |
| Proteins | -0.0490 (-0.0713, -0.0266) | 0.00002 |  | -0.0485 (-0.0709, -0.026) | 0.00002 |
| Potassium | -0.0007 (-0.0012, -0.0003) | 0.00200 |  | -0.0006 (-0.001, -0.0001) | 0.01308 |
| Phosphorus | -0.0016 (-0.0031, -0.0001) | 0.03389 |  | -0.0012 (-0.0027, 0.0004) | 0.13394 |
| AHEI | -0.0086 (-0.0300, 0.0128) | 0.43190 |  | 0.0077 (-0.0144, 0.0297) | 0.49470 |
| Mediterranean | 0.0034 (-0.1529, 0.1596) | 0.96635 |  | 0.0779 (-0.0816, 0.2375) | 0.33817 |
| DASH | 0.0047 (-0.0481, 0.0575) | 0.86157 |  | 0.0438 (-0.0104, 0.0980) | 0.11289 |

*Abbreviations*: PGS, polygenic score; eGFR, estimated glomerular filtration rate; 95%CI, 95% confidence interval.

**Adjustments* are:

- Minimally adjusted: Adjusted for age, sex, and the first 10 genetic principal components
- Fully adjusted: Minimally adjusted + BMI, physical activity, smoking habits, and education.

** *Dietary exposure*: Proteins, Potassium, Phosphorus, AHEI, Mediterranean, DASH

**Table S7:** Results of the regression analyses assessing the association between proteins, PGS, and their interaction with eGFRcrea, comparing three modelling approaches: linear regression (LM), linear regression including principal components (LM + PCs), and linear mixed‑effects models with a kinship random effect (LMM with kinship). The table presents β coefficients, standard errors, and p‑values for each term across all six models. Models were fitted in R using the *gam* function of the *mgcv* package (v1.8-40.) for non-linear effects, with non-linear main and interaction effects extracted via the *predict* function, and LMMs fitted using *lmekin* from the *coxme* package (v2.2-22).

| Minimally adjusted models* | | | | | | | | | | | |
| --- | --- | --- | --- | --- | --- | --- | --- | --- | --- | --- | --- |
|  | LM | | |  | LM+PCs | | |  | LMM (kinship matrix) | | |
|  | β | SE | p |  | β | SE | p |  | β | SE | p |
| PGS | 1.00 | 0.08 | <2e‑16 |  | 0.99 | 0.08 | <2e‑16 |  | 0.97 | 0.08 | <2e‑16 |
| Proteins | 1.03 | 0.22 | 2.06e‑06 |  | 1.03 | 0.22 | 2.00e‑06 |  | 0.95 | 0.21 | 7.1e‑06 |
| Proteins:PGS | 1.38 | 0.30 | 3.86e‑06 |  | 1.38 | 0.30 | 3.58e‑06 |  | 1.35 | 0.29 | 2.5e‑06 |
| Fully adjusted models* | | | | | | | | | | | |
|  | LM | | |  | LM+PCs | | |  | LMM (kinship matrix) | | |
| Term | β | SE | p |  | β | SE | p |  | β | SE | p |
| PGS | 0.98 | 0.08 | <2e‑16 |  | 0.98 | 0.08 | <2e‑16 |  | 0.96 | 0.08 | <2e‑16 |
| Proteins | 1.03 | 0.22 | 2.33e‑06 |  | 1.02 | 0.22 | 2.58e‑06 |  | 0.94 | 0.21 | 1.1e‑05 |
| Proteins:PGS | 1.37 | 0.30 | 4.58e‑06 |  | 1.36 | 0.30 | 4.53e‑06 |  | 1.34 | 0.29 | 2.7e‑06 |

*Abbreviations*: PGS, polygenic score; eGFR, estimated glomerular filtration rate; *LM, linear regression model; LM + PCs, linear regression including the first 10 genetic principal components; LMM* *with kinship, linear mixed‑effects model including a kinship random effect.*

** Adjustments* are:

- Minimally adjusted: Adjusted for age and sex
- Fully adjusted: Minimally adjusted + BMI, physical activity, smoking habits, and education.

**Table S8**: Association of residualized phosphorus intake with eGFRcrea in minimally adjusted and fully adjusted models. The table reports estimated degrees of freedom (df) for smooth terms and corresponding p-values for the minimally adjusted models.

|  | Minimally adjusted model* | | Fully adjusted model* | |
| --- | --- | --- | --- | --- |
|  | edf | p-value | edf | p-value |
| PGS | 1.006 |  | 1.007 | <0.0001 |
| Phosphorus (pre-adjusted^) | 1 | 0.245 | 1 | 0.0532 |
| PGS:Phosphorus (pre-adjusted^) | 2.887 | 0.365 | 3.67 | 0.225 |

^ Pre-adjusted by using residualization, removing the effect of protein intake

**Adjustments* are:

- Minimally adjusted: Adjusted for age, sex, and the first 10 genetic principal components
- Fully adjusted: Minimally adjusted + BMI, physical activity, smoking habits, and education.

**Table S9**: Results of the GAM analysis assessing the association between PGS and dietary exposures^ with eGFRcrea, comparing two approaches: the ad hoc PGS and PRS-CS. The table reports estimated degrees of freedom (df) for smooth terms and corresponding p-values for the minimally adjusted models.

|  | Minimally adjusted* | | | | Fully adjusted* | | | |
| --- | --- | --- | --- | --- | --- | --- | --- | --- |
|  | PGS - primary analysis | | PGS_eGFRcrea_ | | PGS - primary analysis | | PGS_eGFRcrea_ | |
|  | df | p-value | df | p-value | df | p-value | df | p-value |
| PGS | 1.00 | <0.0001 | 2.59 | <0.0001 | 1.00 | <0.0001 | 2.62 | <0.0001 |
| Proteins | 1.00 | <0.0001 | 1.01 | <0.0001 | 1.00 | <0.0001 | 1.00 | <0.0001 |
| Proteins:PGS | 4.93 | 0.0298 | 1.01 | 0.0281 | 4.82 | 0.0335 | 1.00 | 0.0214 |
| PGS | 1.00 | <0.0001 | 2.62 | <0.0001 | 1.00 | <0.0001 | 2.64 | <0.0001 |
| Potassium | 1.00 | 0.0010 | 1.00 | 0.0039 | 1.00 | 0.0061 | 1.00 | 0.0160 |
| Potassium:PGS | 1.00 | 0.6783 | 1.00 | 0.9202 | 1.00 | 0.7064 | 1.00 | 0.9939 |
| PGS | 1.01 | <0.0001 | 2.62 | <0.0001 | 1.01 | <0.0001 | 2.63 | <0.0001 |
| Phosphorus | 1.00 | 0.0241 | 1.00 | 0.0496 | 1.00 | 0.0864 | 1.00 | 0.1374 |
| Phosphorus:PGS | 5.37 | 0.0283 | 1.14 | 0.8861 | 5.46 | 0.0223 | 1.21 | 0.8392 |
| PGS | 1.00 | <0.0001 | 2.63 | <0.0001 | 1.01 | <0.0001 | 2.65 | <0.0001 |
| AHEI | 1.91 | 0.1788 | 1.92 | 0.2062 | 1.54 | 0.3974 | 1.57 | 0.3601 |
| AHEI:PGS | 1.04 | 0.2773 | 1.01 | 0.3502 | 1.01 | 0.2688 | 1.01 | 0.3345 |
| PGS | 1.00 | <0.0001 | 2.60 | <0.0001 | 1.00 | <0.0001 | 2.63 | <0.0001 |
| Med | 1.02 | 0.8872 | 1.01 | 0.9111 | 1.02 | 0.2853 | 1.01 | 0.3692 |
| Med:PGS | 1.00 | 0.2548 | 1.01 | 0.9071 | 1.01 | 0.2187 | 1.01 | 0.9336 |
| PGS | 1.00 | <0.0001 | 2.63 | <0.0001 | 1.00 | <0.0001 | 2.65 | <0.0001 |
| DASH | 2.22 | 0.0606 | 2.25 | 0.0530 | 2.13 | 0.0246 | 2.16 | 0.0227 |
| DASH:PGS | 1.01 | 0.4705 | 1.03 | 0.3225 | 1.01 | 0.5379 | 1.04 | 0.3495 |

*Abbreviations*: GAM, generalized additive model; PGS, polygenic score; eGFR, estimated glomerular filtration rate; df, degrees of freedom;

^^^*Dietary exposure*: Proteins, Potassium, Phosphorus, AHEI, Mediterranean, DASH

*Adjustments* are:

- Minimally adjusted: Adjusted for age, sex, and the first 10 genetic principal components
- Fully adjusted: Minimally adjusted + BMI, physical activity, smoking habits, and education.
